# Evaluation of secondary dentin formation for forensic age assessment by means of semi-automatic segmented ultrahigh field 9.4 T UTE MRI datasets

**DOI:** 10.1007/s00414-020-02425-7

**Published:** 2020-09-17

**Authors:** Maximilian Timme, Jens Borkert, Nina Nagelmann, Andreas Schmeling

**Affiliations:** 1 Institute of Legal Medicine, University Hospital Münster, Röntgenstraße 23, 48149 Münster, Germany; 2 Department of Clinical Radiology, Translational Research Imaging Center (TRIC), University Hospital Münster, Albert-Schweitzer-Campus 1, 48149 Münster, Germany

**Keywords:** Age assessment, Secondary dentin formation, MRI, Dental age, Ultrashort time echo

## Abstract

Dental methods are an important element of forensic age assessment of living persons. After the development of all the teeth, including third molars, is completed, degenerative characteristics can be used to assess age. The radiologically detectable reduction of the dental pulp cavity has been described as such a feature. We investigated the suitability of ultrahigh field 9.4 T ultrashort time echo (UTE) magnetic resonance imaging (MRI) for the evaluation of pulp cavity volume in relation to the total tooth volume in 4 extracted human teeth. The volume calculations were performed after semi-automatic segmentation by software AMIRA using the different intensities of the structures in the MRI dataset. The automatically selected intensity range was adjusted manually to the structures. The visual distinction of pulp and tooth structure was possible in all cases with in-plane resolution < 70 μm. Ratios of tooth/pulp volume were calculated, which could be suitable for age estimation procedures. Intensity shifts within the pulp were not always correctly assigned by the software in the course of segmentation. 9.4 T UTE-MRI technology is a forward-looking, radiation-free procedure that allows the volume of the dental pulp to be determined at high spatial resolution and is thus potentially a valuable instrument for the age assessment of living persons.

## Introduction

Forensic age assessment can provide clarity in the case of missing or doubtful age information and thus contribute to the execution of constitutional proceedings. In forensic age estimation, the grade of development of various skeletal and dental features is determined and compared with reference values [1]. Once tooth development is complete, only degenerative dental characteristics can be used for age assessment [2–6]. One such feature has long been known to be the reduction of the dental pulp due to secondary dentine formation [3, 7–10].

The physiological background of secondary dentin formation is that odontoblasts continue to produce dentin continuously after the completion of tooth development [11–13]. This so-called secondary dentine is added to the dentine facing the pulp, which reduces the volume of the pulp cavity during life. Today it is considered that secondary dentin formation is a cellular synthesis process independent of external factors [14]. The tertiary dentine must be distinguished from the secondary dentine. Tertiary dentin is a reaction of the odontoblasts to various pathological processes usually in the context of caries or wear [15, 16]. Tertiary dentine formation does not correlate with age [17, 18]. The differentiation between tertiary and secondary dentine has so far only been possible histologically, which is why great importance must be attached to not determining the formation of secondary dentine on pathologically influenced teeth for the propose of forensic age assessment [19]. In contrast, the feature seems to be independent, e.g., of orthodontic treatment [20].

In the recent past, increasing numbers of publications have reported an approach to calculate the actual volume of the dental pulp or a quotient of the volume of the hard tooth substance and the pulp [21–26]. These methods are based on the cone-beam CT (CBCT) for imaging. CBCT creates a 3D dataset from which the reconstruction of the structures is possible [9]. However, the CBCT is associated with a higher radiation exposure than the conventional dental radiological methods [27]. This fact is critical for this method.

A new development is the use of magnetic resonance imaging (MRI) technology in dental imaging, as a radiation-free method [28–30]. Consequently, the technology has already been used for dental age assessment [31–33]. However, imaging of hard tissues like the bone or teeth is still challenging because of their limited water content and solid structure [34]. Due to this special challenge, sequences that are sensitive despite ultrashort T2 relaxation can be used to analyze these tissues at relatively high spatial resolution and with a high signal to noise ratio [35]. One MRI sequence that meets these requirements is ultrashort time echo [36–39].

The aim of the present study was to examine whether UTE-MRI is suitable for visualizing tooth hard tissue and dental pulp enabling calculation of a quotient for age assessment applicable on the different types of human teeth.

## Materials and methods

This study was approved by the responsible ethics committee (2017-215-f-S). All donors of teeth signed a consent form for the use of their teeth for scientific purpose.

For the present study, a total of 4 extracted human teeth were examined by MRI. The teeth came from 2 males and 1 female aged 48, 54, and 78 years, respectively. All the teeth were extracted for medical indication. Only the teeth which did not show any visible pathological lesions were used for the examinations. The teeth were placed in 70% ethanol immediately after extraction. The teeth were an anterior tooth (FDI 11), a canine tooth (FDI 23), one premolar (FDI 14), and one molar (FDI 26).

Approximately 24 h before the MRI examinations, the teeth were taken from the alcohol, rinsed, and embedded in 1% agarose in a 5-ml falcon tube and stored at 4 °C overnight.

MRI was performed on a 9.4 T Bruker Biospec 94/20 (Bruker BioSpin GmbH, Ettlingen, Germany) equipped with a 35-mm quadrature birdcage coil (Rapid Biomedical, Rimpar, Germany). The falcon tube with the embedded tooth was put directly into the 35-mm micro-coil and fixed with paper towels in the middle of the coil to avoid movement artifacts and to position the sample in the isocenter. 3D UTE sequence was used with the following parameters: time to repetition, 8.0 ms; time to echo, 0.020 ms; flip angle, 5°; averages, 4; scan time, 1 h 12 min; number of projections, 134,526; polar undersampling, 1.52; and Matrix, 256. Due to different types of examined teeth, field of view and spatial resolution had to be adjusted for each tooth. Table 1 shows the field of view and spatial resolution values for each tooth.

**Table 1:** Measurement data by tooth

Tooth (FDI)	Field of view (mm^3^)	Spatial resolution (μm^3^)
11	17 × 30 × 17	66 × 117 × 66
23	17 × 35 × 17	66 × 137 × 66
14	17 × 30 × 17	66 × 117 × 66
26	17 × 31 × 17	66 × 121 × 66

3D reconstruction of MRI datasets was performed with AMIRA software (Visage Imaging GmbH, Berlin, Germany).

The semi-automatic segmentation was performed with the so-called “magic wand tool” of AMIRA software. The “magic wand tool” bases the selection off voxel color gradient. Thus, transferred to the MRI, the selection is based on the different intensities of the tissues. The automatically selected intensity range was adjusted manually to the structures. The range was adjusted until the area of the selection visually matched the area of the structure exactly. However, this adjustment of the assignment was only carried out in one slice. For segmentation, the selection made by the tool was not further corrected manually.

MRI data were further analyzed using profile plot analysis tool of ImageJ software (Version 1.50b, Wayne Rasband, National Institute of Health, USA). Signal intensities were reported as arbitrary units (au); values were given as mean.

## Results

UTE-MRI enabled the embedding material, tooth structure, and pulp to be distinguished precisely. The pulp could be traced through the root canals to the apical foramen. A spatial in-plane resolution of 66 μm was achieved for all the teeth. Table 1 shows the specific data measured for each tooth.

**Fig. 1:**
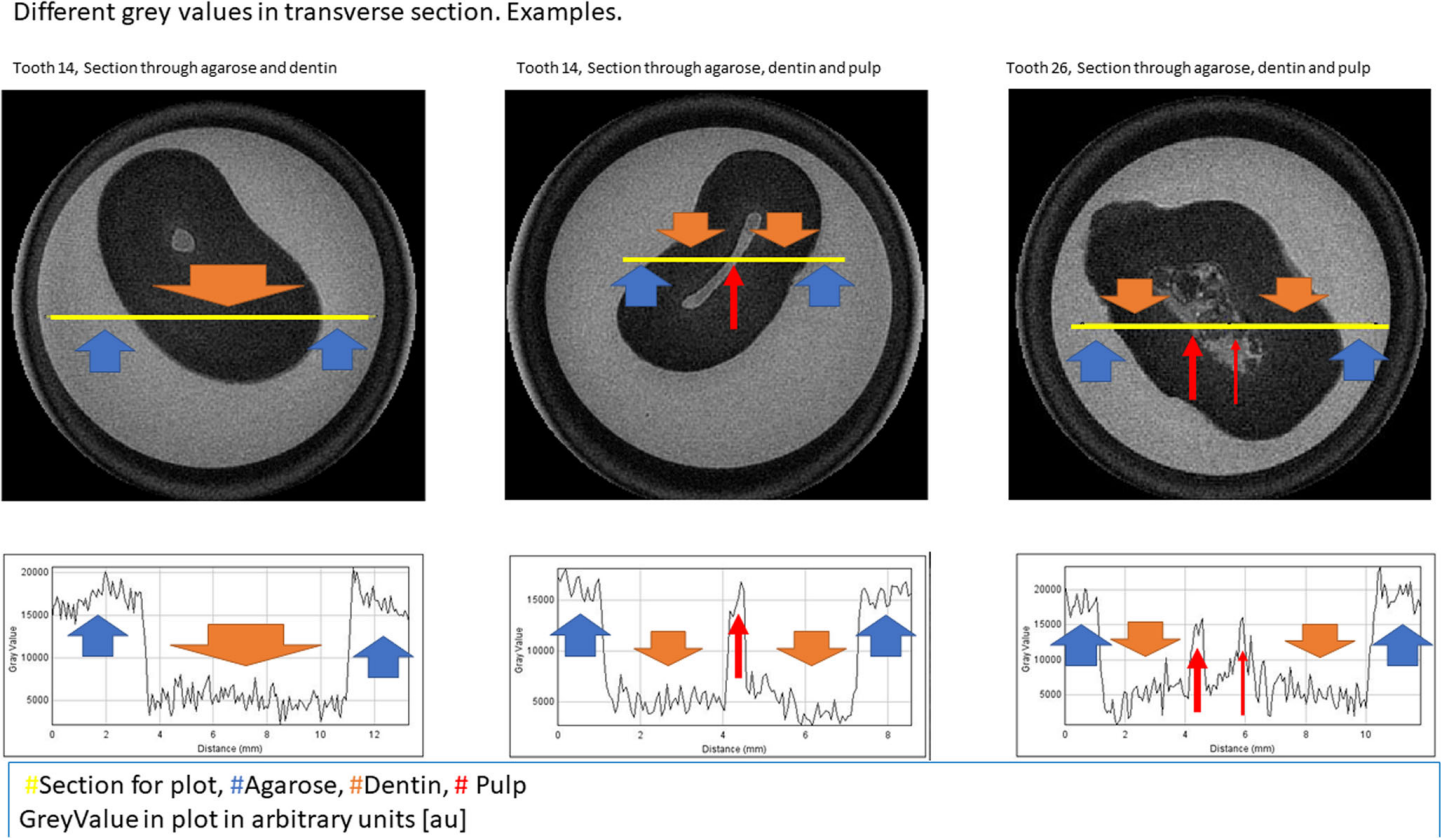
Different grey values in transverse section. Examples

The embedding material showed a signal intensity of mean 16,051 au. In comparison, the hard tooth substance can be clearly distinguished with a signal intensity of mean 5639 au. The pulp showed a signal intensity of mean 13,452 au and is thus in a comparable range with the embedding material (Fig. 1)

In tooth 26, significant intensity changes were observed within the area of the pulp (Fig. 1).

**Fig. 2:**
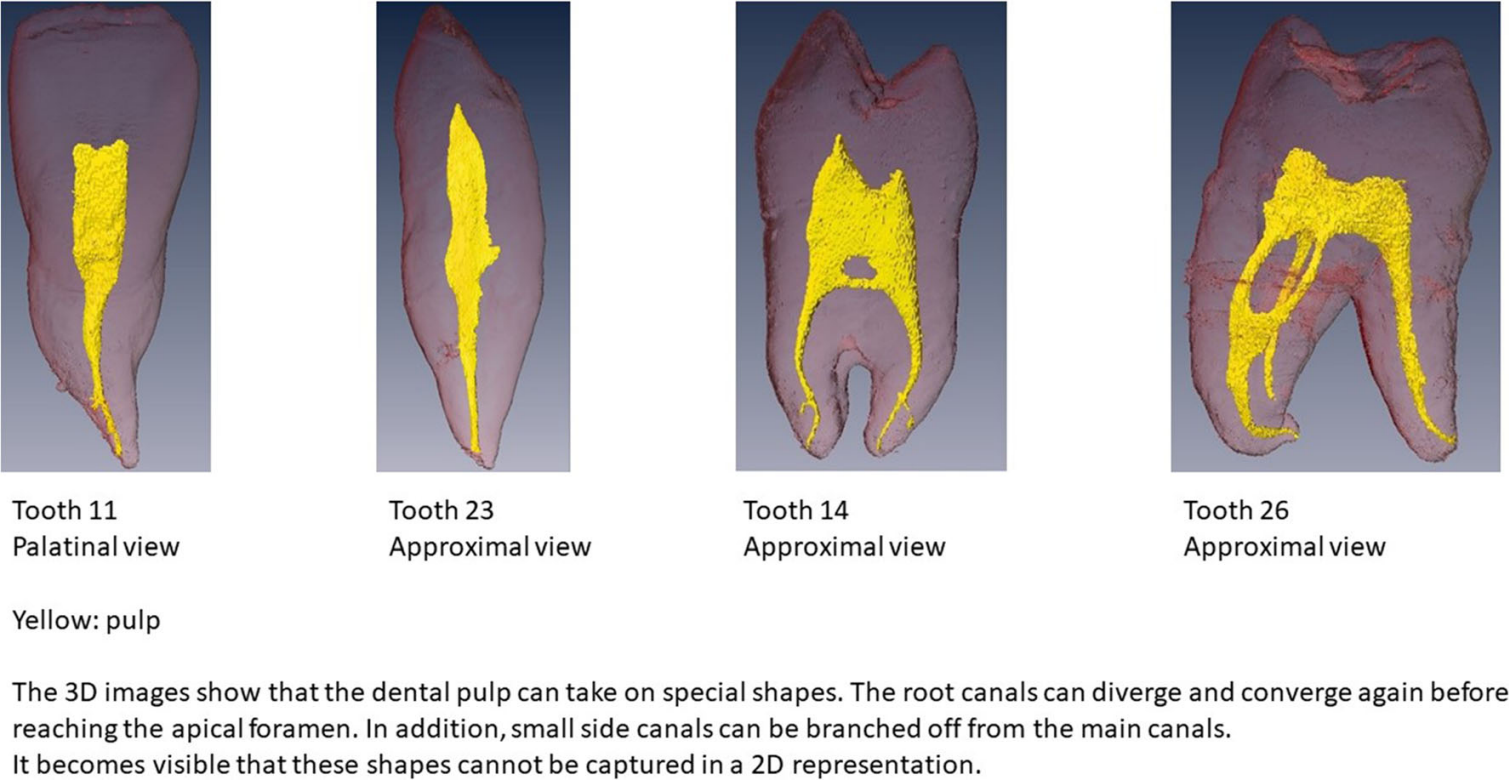
3D reconstructions of all teeth

For all teeth, a 3D reconstruction could be generated, and the volumes could be calculated. Figure 2 shows the special formations the dental pulp can take. In tooth 26, it becomes evident that the pulp, especially the root pulp, can take on special formations. Root canals that physiologically diverge and re-confluence within the root can be found. Table 2 lists the calculated volumes for each tooth. The calculated total volumes were between 605.80 and 992.92 mm^3^ for 11 and 26, respectively.

**Table 2:** Volume calculations and quotients per tooth

Tooth [FDI]	11	14	23	26
Volume total (mm^3^)	605.80	762.37	723.11	992.92
Volume pulp (mm^3^)	12.74	15.79	12.70	29.29
Quotient pulp/total (%)	2.1	2.07	1.76	2.95

The values for the pulp volumes ranged from 12.70 (tooth 23) to 29.29 mm^3^ (tooth 26).

Ratios of tooth/pulp volume were calculated and given in Table 2.

## Discussion

The purpose of this study was to examine whether modern MRI technology is suitable for imaging the dental pulp in such quality that a use in dental age assessment is possible. This approach is desirable, since on the one hand the possibilities of three-dimensional methods should be used in age assessment of living individuals, but on the other hand the radiation exposure should be kept as low as possible for reasons of radiation protection.

Using a tomograph with a field strength of 9.4 T and the UTE sequence, technology was applicated which is in the range of what is currently technically possible. In this context, it should be noted that the field strengths of the tomographs, as they are used in the clinical context today, are in the range of 1.5 to 3 T or in exceptions up to 7 T [40, 41]. In addition, UTE technology is not yet widely available. Thus, the present study is a view in the direction of what could be possible in the field of forensic dental imaging in the future.

When measuring the secondary dentine formation for the purpose of age determination, one question is that of the most suitable teeth [22]. In the present study, it could be shown that for all human tooth types (front, canine, premolar, molar), the detailed imaging of the pulp up to the apical foramen is possible at high resolution. Therefore, this method does not necessarily have to be limited to single-rooted teeth. The challenge in the future will rather be the detection of pathological influences on secondary dentine formation. These teeth should be excluded from the age estimation in principle.

The spatial resolution is described as one of the most important parameters that objectively determine the image quality, especially in dental imaging, where fine details often need to be displayed [42]. With the selected parameters, an in-plane resolution of 66 μm^3^ could be achieved in the present study. Thus, comparable values to those obtained in CBCT investigations could be achieved [43]. However, it should be noted that very high resolutions in CBCT are also associated with higher radiation exposure.

Further technical innovations in the field of segmentation will be necessary in the future, as a comparable study with CBCT has shown that the segmentation procedure can influence the results [44]. However, it is not clear in this context how the segmentation methods can be combined with MRI technology.

Due to the research question, it was decided not to relate the pulp volume to the age of the tooth donor. This correlation has been examined in principle and, with reference to new MRI parameters, should be verified in large reference studies.

In addition, special statistical methods were not used because of the small number of cases. In view of the research question of the study, no additional knowledge gain was expected from the use of special statistical methods.

## Outlook and conclusions

The promising results of the present study should be verified on a larger number of cases in the future. For this purpose, the age of the persons must also be correlated with the results of the volume measurements. This can also be used to determine which tooth correlates best with age. For the method of semi-automatic segmentation presented in this paper, the intra- and interrater agreement should be determined.

In addition, the accuracy of semi-automatic segmentation should be compared with manually corrected or fully automatic segmentation.

Furthermore, the method should also be tested against the current gold standard of CBCT in the future.

As soon as technically possible, the results of this in vitro approach should be transferred to in vivo.

All in all, 9.4 T UTE-MRI is a suitable, radiation-free tool for imaging tooth and pulp with a high spatial resolution for dental age assessment quite comparable with CBCT data. Further technical developments as well as scientific studies are necessary until practical application.
